# Friend, Not Foe: Unveiling Vector-Bacteria Symbiosis and Its Utility as an Arboviral Intervention Strategy in the Philippines

**DOI:** 10.3389/fcimb.2021.650277

**Published:** 2021-06-29

**Authors:** Shaira Limson Kee, Myles Joshua Toledo Tan

**Affiliations:** ^1^ Department of Natural Sciences, University of St. La Salle, Bacolod, Philippines; ^2^ Department of Chemical Engineering, University of St. La Salle, Bacolod, Philippines

**Keywords:** vector-borne disease control, dengue, Philippines, Wolbachia, symbiosis, arbovirus, mosquito, biocontrol

## Introduction

Humans have long studied the ecological symbiosis of organisms primarily from an anthropocentric perspective. Among these investigations are the symbiosis of bacteria and arthropods. Studies conducted in relation to this usually lean toward subjects of pathogen transmission, cure, and prevention. However, Bourtzis and Zchori-Fein, editors of *Manipulative Tenants: Bacteria Associated with Arthropods*, discuss the diversity of the symbiosis that occurs between bacteria and arthropods. They also explain that despite common negative associations between them, microbial interaction with its vector can also benefit eukaryotic organisms, including humans. In fact, some symbiotic relationships that involve bacteria are essential to the survival of arthropods. One major benefit that this relationship offers to increase the survival of humans is its purpose as a biocontrol strategy aimed toward minimizing arboviruses. This presents a significant benefit as the alarming trend of insecticide-resistant mosquitoes intensifies due to gradual adaptation to conventional control measures (Brathwaite Dick et al., 2012; Marcombe et al., 2012). Further research into symbiosis will greatly benefit countries like the Philippines whose commonly applied intervention strategy for dengue involves the use of insecticides and manual elimination of mosquito breeding sites.

## Functional Symbiosis

Symbiosis, as defined by Heinrich Anton de Bary (1878), is the living together of organisms originating from separate species (Oulhen et al., 2016). It occurs in multivariate forms and can be either endosymbiotic, like between *Pseudotrichonympha* spp. in the gut of subterranean termites and the nitrogen-fixing endosymbiont *Azobacteroides pseudotrichonymphae*, or ectosymbiotic, like between *Devescovina* spp. in the gut of dry-wood termites and the nitrogen-fixing cell surface-attached ectosymbiont *Armantifilum devescovinae* (Strassert et al., 2010; Desai and Brune, 2012). Symbionts, whether primary or secondary, play different roles in the lives of their hosts, some of which supplement metabolic functions, determine alterations, manipulate host reproduction and protect against pathogens (Zchori-Fein and Bourtzis, 2012). These aid in increasing an organism’s fitness and enable adaptive radiation to further increase its ecological niche. This is the reason why mammalian systems are more complex, and why microbial diversity is greater among mammals of the same species than among insects of the same species. However, greater diversity can be observed among different insect species than among different mammal species. This is observed to be a consequence of the greater variations found in the diet and gut physiology of insects when compared with those of mammals (Douglas, 2011). The co-existence of these organisms have been apparent in nature for a long time. As a consequence of this long history of association, a dependency on the relationship has been established in many instances such that deprivation of the symbiont results in the inability of the organism to grow and reproduce (Douglas, 2007).

## Implications of Transmission

The functions provided by bacteria to their hosts vary among species. This diversity among microbes was thought to arise from differences in lineage, but due to recent developments, it was discovered that these differences may not be as large as initially hypothesized. Particular genera of bacteria are constantly found in hosts that are distantly related (Zchori-Fein and Bourtzis, 2012). These are potentially effects of different bacterial transmissions that occur either vertically or horizontally. Vertical transmission involves parent-to-offspring exchange of microbes due to their presence in the reproductive organs of the host (Ricci et al., 2012). On the other hand, horizontal transmission, which occurs between individuals of different species, offers a topic of interest. This cross-colonizing capability of selected bacteria among different species can be witnessed in *Spiroplasma, Arsenophonus, Asaia* and *Wolbachia* groups (Fukatsu et al., 2001; Gómez-Valero et al., 2004; Marzorati et al., 2006; Wille and Hartman, 2009). These resilient individuals have been found to have evolved mechanisms that allow them to colonize and survive different immune systems. In fact, several studies have shown a comparison of vector competence of both aseptic (absence of endogenous bacterial flora) and septic mosquitoes. The results indicated that gut microbes can potentially reduce *Plasmodium* infection among mosquitoes and that aseptic mosquitoes have an increased susceptibility (Pumpuni et al., 1996; Gonzalez-Ceron et al., 2003; Dong et al., 2009). Along with that, the absence of *Wigglesworthia*, which is an obligate symbiont of flies, was also observed to have resulted in an increased susceptibility to parasitic infections (Pais et al., 2008). With that, microbiota can either directly or indirectly affect the vectorial competence of their hosts through interactions with parasites either by inhibitory bioactivity functions of their secretions, or by host immune system stimulation that signals the clearing of pathogenic microbes, respectively (Weiss and Aksoy, 2011; Ricci et al., 2012). Aside from this, zoonotic diseases are also influenced by changes in topography, deforestation, and agricultural land conversion. These anthropogenic changes that are commonly witnessed in the Philippines are key drivers for the proliferation of mosquitoes and their associated pathogens (Walsh et al., 1993; Guerra et al., 2006; Yasuoka and Levins, 2007; Jones et al., 2008; Olson et al., 2010; Fornace et al., 2016). An essential note to focus on is that they offer interesting implications in understanding the response of the body’s immune system during host invasion. Further research will help us understand vector-borne pathogens better.

## Biocontrol Strategy Against Arboviruses

In the public view, increased arthropod fitness and resilience to different internal systems due to microbial symbiosis does not offer a positive subject because arthropods, primarily mosquitoes, are known as harborers of pathogens. However, this can, in fact, be a useful solution to humanity’s alarming arboviral issues through the use of biocontrol strategies. Abundant research on reproductive parasites over the past 25 years have been centered on Wolbachia, an intracellular bacterium that is dependent on the cytoplasmic environment of its host (Zchori-Fein and Bourtzis, 2012). With that, Wolbachia was discovered to induce cytoplasmic incompatibility in their infected hosts. This biological process alters the reproductive functions of the host by inducing high embryonic mortality, thereby decreasing the number of viable offspring (Hoffmann et al., 1986; Vavre et al., 2000; Tram and Sullivan, 2002; Bordenstein and Werren, 2007). Research on bacterial symbionts is not solely concentrated on Wolbachia. In fact, *Pantoea agglomerans* is a bacterial symbiont of the *Anopheles* mosquito that was transformed to express anti-*Plasmodium* activity through effector proteins pelB or hlyA. Aside from this, symbiont bacteria *Rhodococcus rhodnii*, *Asaia* and *Pantoea* are also potentially effective for symbiotic control strategies (Ricci et al., 2012). Years of research and field experimentation have proven that bacteria have the ability to restrict arbovirus transmission. This serves as a promising intervention to reduce the incidence of vector-borne diseases (Sinkins, 2004; Kambris et al., 2009; McMeniman et al., 2009; Moreira et al., 2009). This can benefit countries like the Philippines (~400,000 reported cases in 2019 according to the World Health Organization) that suffer frequent dengue outbreaks. Another interesting fact is that through application of highly sensitive polymerase chain reaction (PCR) methods accompanied by DNA hybridization and sequence analysis techniques, it was revealed that Wolbachia infection is evident in up to 76% of all arthropod species (Jeyaprakash and Hoy, 2000). This means that it is common worldwide. Its widespread population emphasizes the adaptability of these microorganisms. In fact, this extensive distribution could be attributed to the evolutionary success induced by the symbiotic relationships between mosquitoes and several microorganisms (Ricci et al., 2012). This versatility can be manipulated to benefit future intervention strategies. To add, this prevalence in nature means that it can be applied anywhere around the globe as it is not climatically restricted. Taking these factors into consideration, increasing the limited research on Wolbachia in the Philippines and integrating it into the national intervention strategy will likely produce better results than current approaches.

## Discussion

The wide array of symbiotic relationships displayed by arthropods and their symbionts are evidence that these associations are relatively ubiquitous and ever-evolving in nature. For instance, organisms involved in parasitism are observable in all major clades of eukaryotes. Moreover, these parasites were found to share a common ancestry with their free-living relatives. This suggests that convergence through occupying similar environmental niches and reductive evolution played a huge part in their evolution (Walochnik and Duchêne, 2016). With that, humans can utilize this interdependence and interrelatedness of organisms to innovate new forms of symbiosis, and thereby, tackle existing health issues such as arboviral diseases without resorting to eradicative measures. Just like how bacteria thrive by exchanging nutrients that are inadequately represented in the diet of their eukaryotic partners, humans can participate in this type of symbiosis, as well. By aiding these Wolbachia-infected mosquitoes, they can infect other vectors through horizontal and vertical transmissions and thereby increase their population (Laven, 1967; Zabalou et al., 2004; McMeniman et al., 2009). This can lead to greater chances of preventing the transmission of arboviral diseases (e.g. dengue, Chikungunya, Zika), and malaria, that have been proven to be inhibited by Wolbachia infection of their vectors (Moreira et al., 2009; Caragata et al., 2016).

Further knowledge on these topics opens the less ventured idea that pathogenic infections are not the sole relationship that humans share with arthropods and that mutualistic associations are also prevalent. This is not a particularly new idea since human symbiocosm has existed for many years. A good example would be bacterial species more commonly classified as “good bacteria" that live permanently through commensal or mutualistic interactions with human hosts, especially in the digestive tract. The fact that these organisms are necessary enough to have aided in the continuation of the existence of some arthropods for more than 275 million years is a point to ponder. Perhaps we, humans, may benefit from this symbiosis for our own survival. *Manipulative Tenants: Bacteria Associated with Arthropods*, along with its findings from years of research and the increasing success of field experimentation in this area, is solid proof that an efficient action plan that would utilize Wolbachia as an intervention strategy against arboviruses, particularly dengue, must be applied and disseminated throughout the Philippines in order to mitigate the concurrent issues, if not eradicate them once and for all.

## Author Contributions

Conceived the work: MT. Drafted the article: SK. Critically revised the manuscript: MT. All authors contributed to the article and approved the submitted version.

## Conflict of Interest

The authors declare that the research was conducted in the absence of any commercial or financial relationships that could be construed as a potential conflict of interest.
